# Mitral Leaflet Separation Index in Assessing the Severity of Mitral Stenosis

**DOI:** 10.5402/2011/768097

**Published:** 2011-05-19

**Authors:** Joby K. Thomas, T. M. Anoop, Gailin B. Sebastian, Kim George, Raju George

**Affiliations:** ^1^Department of Cardiology, Kottayam Medical College, Kerala 686008, India; ^2^Department of Medicine, Kottayam Medical College, Kerala 686008, India

## Abstract

Determining the severity of mitral stenosis (MS) is important for both prognostic and therapeutic reasons. The mitral valve area (MVA) can be measured by planimetry, pressure half-time, continuity equation, and proximal isovelocity surface area methods. In this study, we propose a novel yet simple, independent measure of MS severity–the mitral leaflet separation (MLS) index. This new index could be a useful surrogate measure of the MVA. This index would also help when there is a discrepancy between severities of MS estimated by existing methods, in the presence of atrial fibrillation and in the presence of mitral regurgitation.

## 1. Introduction

Rheumatic mitral stenosis is an acquired progressive valvular heart disease characterized by diffuse thickening of the mitral leaflets, fusion of the commissures, and shortening and fusion of the chordae tendineae, which occur as a sequel to acute rheumatic fever. Determining the severity of mitral stenosis (MS) is important for both prognostic and therapeutic reasons. Two-dimensional (2D) Doppler echocardiography is presently the gold standard method for assessment of severity of MS [1]. The mitral valve area (MVA) can be measured by planimetry, pressure half-time (PHT), continuity equation, and proximal isovelocity surface area methods [2, 3]. Direct measurement of MVA by planimetry is accurate but is highly operator dependent and sometimes laborious. The reliability of the pressure half-time method is affected by changes in preload or left ventricular compliance. The transmitral gradient is also well correlated with MS severity. Transmitral gradient and continuity equation depend on transvalvular flow and may be affected by cardiac output and presence of mitral regurgitation.

In this study, we evaluate a novel yet simple, independent measure of MS severity—the mitral leaflet separation (MLS) index. This new index could be a useful surrogate measure of the MVA. 

## 2. Methods

Consecutive patients of all ages and both sexes with rheumatic mitral stenosis who underwent echocardiography at Medical College Hospital, Kottayam from January 1st to june 30th 2008 were enrolled for the study. Patients with suboptimal images and/or heavy mitral valvular calcification precluding the accurate measurement of cuspal separation were excluded from the study. 

In the study population, the mitral valve area was estimated by the standard 2D echo planimetry and pressure half-time methods. The MLS index was estimated by measuring the maximal separation of tip of the mitral leaflets in end diastole in parasternal long axis (PLAX) view and in apical 4-chamber view (A4C). For patients in sinus rhythm, three measurements were obtained in PLAX and A4C view each. A mean of this was taken as MLS index. For patients in Atrial fibrillation, five measurements were taken in PLAX view and five measurements in A4C view. A mean of this was considered as the MLS index.

MLS index was compared with MVA assessed by planimetry and PHT. Severe MS was defined as MVA of 1 cm^2^ or less by planimetry or pressure half-time. Moderate MS was defined as MVA between 1 cm^2^ and 1.5 cm^2^ by planimetry or pressure half-time method. Mild MS was defined as an MVA of more than 1.5 cm^2^ by planimetry or pressure half-time. Echocardiographic parameters taken were mitral valve area from 2D and PHT, mitral mean and peak gradients, left atrial (LA) size, left ventricular (LV) size, ejection fraction (EF), and cuspal separation in PLAX and A4C view. 

### 2.1. Cuspal Separation Measurement

The maximal separation of the mitral valve leaflet tips was measured from inner edge to inner edge in end diastole in the parasternal long-axis and apical 4-chamber views. These two parameters were averaged to yield the mitral leaflet separation index. 

### 2.2. Statistical Analysis

Linear regression analysis was used to correlate MLS index against MVA by planimetry and PHT method. The MLS index for mild, moderate, and severe MS was analysed using analysis of variance to determine if the index could differentiate categories of MS. The value of MLS index which predicted mild and severe MS with best sensitivity and specificity was determined by receiver operating characteristics curve analysis. All statistical analysis was done using SPSS 11 software for windows XP. 

## 3. Results

**Figure 1:**
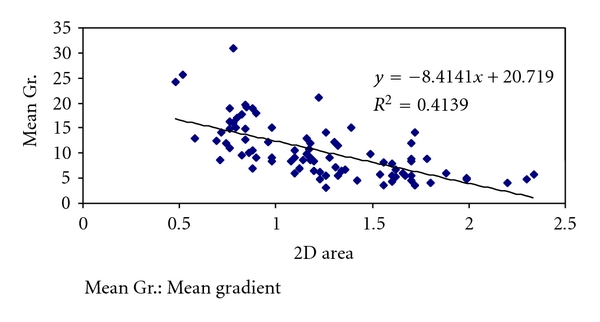
Mean gradient and MVA.

Of 87 patients studied, 19 were males and 68 were females. Age of patients ranged from 19 to 73 years. LA size ranged from 3.2 to 6.5 cm. The Mean LA size was −4.45 cm. There was a strong inverse correlation with cuspal separation (*r* = −0.477) and LA size. LV size ranged from 2.7 to 5.9 cm. Mean LV size was 4.54 cm. There was no correlation between LV size and cuspal separation. EF ranged from 38 to 79%. Mean EF was 67.97%. There was a strong correlation (*r* = 0.3) between EF and cuspal separation. Mean gradient ranged from 3.4 to 32.1 mm of Hg. Average mean gradient was 9.79 mm of Hg. There was a strong inverse correlation of the cuspal separation (*r* = −0.52) with mean gradient. There was also inverse correlation with mean gradient and area detected by planimetry (*r* = −0.643) (Figure 1). 

### 3.1. MVA by Planimetry and Mitral Leaflet Separation

**Figure 2:**
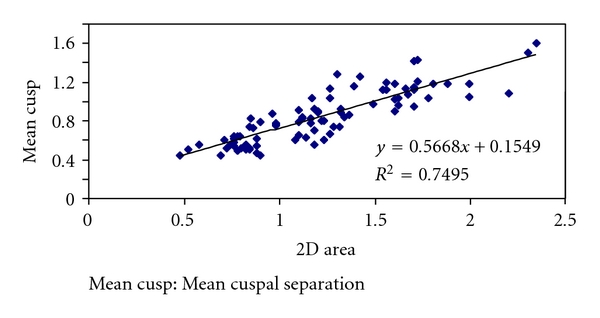
Cuspal separation and MVA.

Mean MVA by planimetry was 1.23 cm^2^. MVA by planimetry ranged from 0.48 to 2.34 cm^2^. There was strong correlation with the mitral leaflet separation (*r* = 0.86) and MVA by planimetry (Figure 2). 

### 3.2. Correlation between MVA and Mitral Leaflet Separation/Mean Gradient

Even though there is a correlation between MVA by planimetry and mean gradient, the correlation between MVA by planimetry and cuspal separation was more statistically significant (*P* < .01) (Table 1). 

The coefficient of correlation between index and mitral valve area by PHT was 0.866, and its *P* value was highly significant (*P* value.000).

### 3.3. Correlation between MVA and Mitral Leaflet Separation/Mean Gradient in Presence of Mitral Regurgitation (MR)

**Figure 3:**
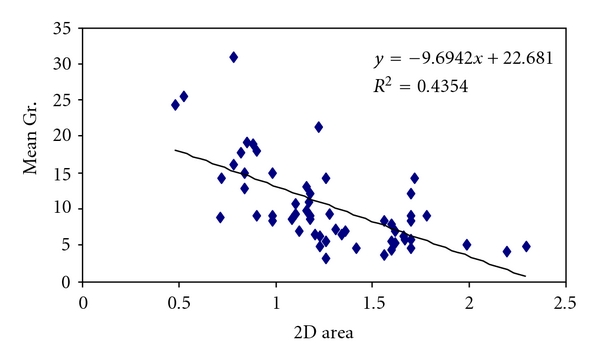
Mean gradient and MVA in presence of MR.

There is a strong inverse correlation between MVA and mean gradient in presence of MR, and the coefficient of correlation was −0.66 (Figure 3).

**Figure 4:**
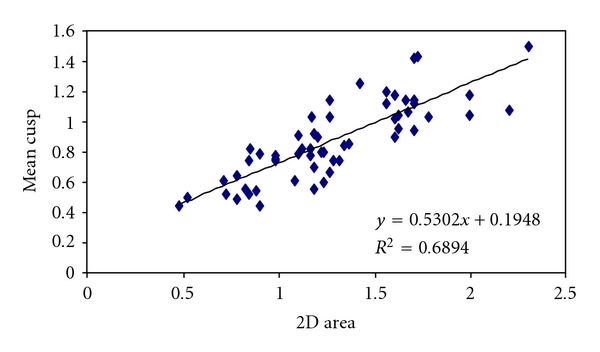
Cuspal separation and MVA in presence of MR.

There was also a significant correlation between MVA and cuspal separation in presence of MR and the coefficient of correlation was 0.83 (Figure 4). But the correlation between MVA and mitral leaflet separation was stronger than the correlation between MVA and mean gradient in presence of significant MR (*P* < .01) (Table 2). 

### 3.4. Correlation between MVA and Mitral Leaflet Separation Index in Presence of Atrial Fibrillation

The total number of patients with atrial fibrillation was 24. The coefficient of correlation between the index with mitral valve area in patients in sinus rhythm was 0.866 (*P* value  .000) and in patients with atrial fibrillation was 0.895 (*P* value  .000), respectively. 

### 3.5. Assessing Severity of MS

**Figure 5:**
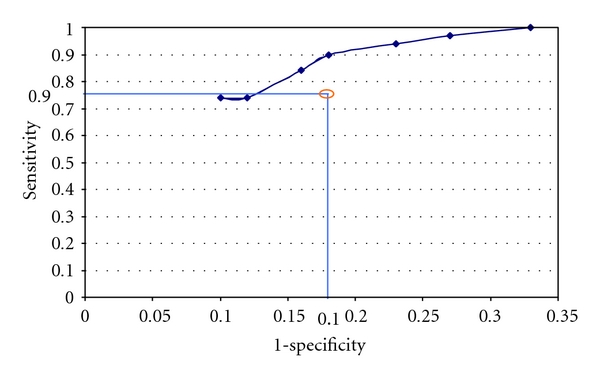
Receiver operating characteristic curve for severe MS.

Using Receiver operating characteristic curve (ROC), mitral leaflet separation less than 7.8 mm can predict severe MS with 90% sensitivity and 82% specificity (Figure 5). 

**Figure 6:**
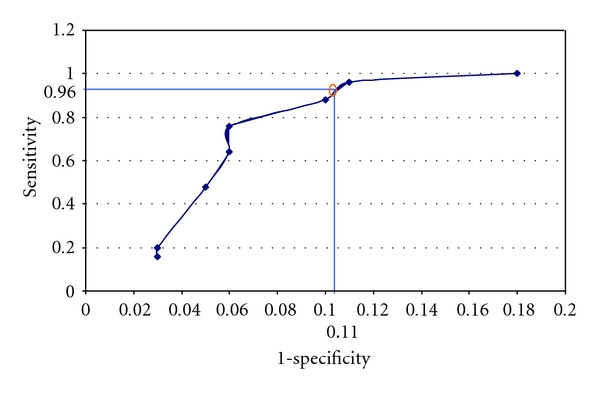
Receiver operating characteristic curve for mild MS.

Using ROC curve, mitral leaflet separation more than 10 mm can predict mild MS with 88% sensitivity and 90% specificity (Figure 6). 

## 4. Discussion

The echocardiography is currently the gold standard method for assessing MS severity. MLS as a measure of MS severity was first proposed by Fisher et al. in 1979 [4]. The study showed a good correlation between maximum diastolic separation distance of the mitral leaflets measured by M-mode and MVA obtained invasively using the Gorlins' equation. Recently, the MLS index, measuring the distance between the tips of the mitral leaflets in parasternal long-axis and four-chamber views, was presented as a reliable measure of MS severity and as a surrogate for MVA [5].

The main advantage of the MLS index is its simplicity and ease of measurement in comparison with planimetry and PHT. It provides a quick estimate of MS severity from standard 2D echocardiographic views without having to resort to tedious measurements as it is technically easy to obtain. The MLS index could be especially useful in situations where there is disagreement between existing methods to the assessment of severity and hemodynamic significance of MS [6]. MLS index can thus be a useful supplement to the existing echo methods for assessment of MS.

MLS index demonstrates an excellent correlation with MVA by planimetry and the pressure half-time method. It is also significantly different for different grades of MS severity, demonstrating high discriminatory ability. It thus reliably differentiated patients with hemodynamically significant MS from those without. The MLS index showed very good correlation with MVA by planimetry in subgroup analysis of patients with AF. In presence of AF, at least five MLS readings in each view were taken and averaged. Thus it remains accurate even in the presence of AF. 

It is a better indicator of MS severity than pressure gradient and can be used as a reliable tool to assess the severity of mitral stenosis in the presence of mitral regurgitation when mean gradient may overestimate the severity of mitral stenosis. Thus it is a better predictor of mitral stenosis severity in the presence of mitral regurgitation. 

## 5. Conclusion

MLS index is a reliable measure of MS severity, which can be used as a an easily obtainable adjunct and sometimes as a surrogate to current methods of assessment but not as a replacement for other echo parameters. This index would also help when there is a discrepancy between severities of MS estimated by existing methods, in the presence of atrial fibrillation and in the presence of mitral regurgitation. 

##  Ethical Approval

The authors fully comply with the ethics in the authorship and publishing of scientific articles [7]. 

##  Funding

There are no sources of funding for this work. 

##  Conflict of interests

There are no competing interests among the authors. 

## Figures and Tables

**Table 1:** Comparison of correlation of MVA with mean gradient and Cuspal separation in MS.

	Gradient	Cuspal separation	*Z* value	*P* value
2D	−0.64	0.86	3.2	<.01

**Table 2:** Comparison of correlation of MVA with mean gradient and cuspal separation in presence of MR.

	Gradient	Cuspal separation	*Z* value	*P* value
2D	−0.66	0.83	3.1	<.01

## References

[1] Nishimura RA, Rihal CS, Tajik AJ, Holmes DR (1994). Accurate measurement of the transmitral gradient in patients with mitral stenosis: a simultaneous catheterization and Doppler echocardiographic study. *Journal of the American College of Cardiology*.

[2] Rifkin RD, Harper K, Tighe D (1995). Comparison of proximal isovelocity surface area method with pressure half-time and planimetry in evaluation of mitral stenosis. *Journal of the American College of Cardiology*.

[3] Lee TY, Hsu T-L, Tseng C-J (2004). Clinical applicability for the assessment of the valvular mitral stenosis severity with Doppler echocardiography and the proximal isovelocity surface area(PISA) method. *Echocardiography*.

[4] Fisher ML, Parisi AF, Plotnick GD (1979). Assessment of severity of mitral stenosis by echocardiographic leaflet separation. *Archives of Internal Medicine*.

[5] Seow SC, Koh LP, Yeo TC (2006). Hemodynamic significance of mitral stenosis: Use of a simple, novel index by 2-dimensional echocardiography. *Journal of the American Society of Echocardiography*.

[6] Raj BSVF, George P, Jose VJ (2008). Mitral leaflet separation index-A simple novel index to assess the severity of mitral stenosis. *Indian Heart Journal*.

[7] Shewan LG, Coats AJS (2010). Ethics in the authorship and publishing of scientific articles. *International Journal of Cardiology*.

